# Let's Stop Trying to Quantify Household Vulnerability: The Problem With Simple Scales for Targeting and Evaluating Economic Strengthening Programs

**DOI:** 10.9745/GHSP-D-17-00291

**Published:** 2018-03-21

**Authors:** Whitney M Moret

**Affiliations:** a FHI 360, Washington, DC, USA.

## Abstract

**Introduction:**

Economic strengthening practitioners are increasingly seeking data collection tools that will help them target households vulnerable to HIV and poor child well-being outcomes, match households to appropriate interventions, monitor their status, and determine readiness for graduation from project support. This article discusses efforts in 3 countries to develop simple, valid tools to quantify and classify economic vulnerability status.

**Methods and Findings:**

In Côte d'Ivoire, we conducted a cross-sectional survey with 3,749 households to develop a scale based on the definition of HIV-related economic vulnerability from the U.S. President's Emergency Plan for AIDS Relief (PEPFAR) for the purpose of targeting vulnerable households for PEPFAR-funded programs for orphans and vulnerable children. The vulnerability measures examined did not cluster in ways that would allow for the creation of a small number of composite measures, and thus we were unable to develop a scale. In Uganda, we assessed the validity of a vulnerability index developed to classify households according to donor classifications of economic status by measuring its association with a validated poverty measure, finding only a modest correlation. In South Africa, we developed monitoring and evaluation tools to assess economic status of individual adolescent girls and their households. We found no significant correlation with our validation measures, which included a validated measure of girls' vulnerability to HIV, a validated poverty measure, and subjective classifications generated by the community, data collector, and respondent. Overall, none of the measures of economic vulnerability used in the 3 countries varied significantly with their proposed validation items.

**Conclusion:**

Our findings suggest that broad constructs of economic vulnerability cannot be readily captured using simple scales to classify households and individuals in a way that accounts for a substantial amount of variance at locally defined vulnerability levels. We recommend that researchers and implementers design monitoring and evaluation instruments to capture narrower definitions of vulnerability based on characteristics programs intend to affect. We also recommend using separate tools for targeting based on context-specific indicators with evidence-based links to negative outcomes. Policy makers and donors should avoid reliance on simplified metrics of economic vulnerability in the programs they support.

## Abstract (precis)

Simple scales developed to measure broad constructs of household economic vulnerability in 3 countries did not accurately measure susceptibility to negative economic outcomes or generate valid classifications of economic status to use for targeting and monitoring and evaluation. We recommend designing tailored monitoring and evaluation instruments to capture narrower definitions of economic vulnerability based on characteristics that economic strengthening programs intend to affect and using separate tools for client targeting based on presence of context-specific “red flag” indicators.

## INTRODUCTION

Economic strengthening programs are intended to help vulnerable households achieve economic stability, often in support of child well-being outcomes in contexts of high HIV prevalence.^1^ The language of economic strengthening is most commonly invoked in the context of projects funded by the United States Agency for International Development (USAID) and the U.S. President's Emergency Plan for AIDS Relief (PEPFAR) for orphans and vulnerable children (OVC) affected by HIV, where interventions are designed to improve economic status in order to help households better access HIV-related services, reduce HIV risk, and improve child well-being. Donors are increasingly demanding that multisectoral programs with economic strengthening components reach the most vulnerable households. Interventions have historically failed to reach such households due to the costs associated with overcoming barriers of social and physical isolation. Donors are also emphasizing the use of “graduation” approaches in economic strengthening programs, where participants exit programs, once they have achieved a set of designated outcomes, making room for new participants. The idea of graduation stems from the need to prevent beneficiaries from depending indefinitely on programmatic inputs, but also the desire to generate sustainable resilience outcomes.

In response to these conditions, practitioners are increasingly seeking data collection tools that will help them target vulnerable households, classify them in terms of economic vulnerability and match them to appropriate economic interventions, monitor participants' status throughout the project, and determine readiness for graduation. To help meet this demand, the ASPIRES (Accelerating Strategies for Practical Innovation and Research in Economic Strengthening) project, funded by USAID and PEPFAR and managed by FHI 360, has experimented with vulnerability assessment methods relevant for economic strengthening projects. Here, we discuss 3 different efforts to quantify and classify individual and household economic status: a large-scale survey in Côte d'Ivoire using rigorous psychometric methods and 2 validation exercises conducted with monitoring and evaluation (M&E) tools developed for different economic strengthening projects in Uganda and South Africa.

This article discusses 3 different efforts to quantify and classify individual and household economic status.

## BACKGROUND ON VULNERABILITY ASSESSMENT

Across disciplines, vulnerability is generally understood as the risk of falling below an accepted benchmark of welfare. Economists typically analyze household survey data using econometric methods to predict economic vulnerability at a population level, answering questions such as^2^:

- What is the extent of vulnerability?
- Who is vulnerable?
- What are the sources of vulnerability?
- How do households respond to shocks?
- What gaps exist between risks and risk management mechanisms?

These data can generate indicators that are useful for policy makers to target resources to geographical regions or households with characteristics associated with economic vulnerability. Economic strengthening practitioners need vulnerability assessment tools that can help them target households and individuals and determine if their vulnerability has decreased during an intervention.

The most intuitive basis for targeting and monitoring economic strengthening interventions is to measure poverty levels. Household poverty, however, can be difficult to measure accurately. One popular tool for doing so is the Progress out of Poverty Index (PPI), a validated, 10-question scorecard that includes indicators derived from national survey data to predict the likelihood that a household falls beneath a given poverty line.^3^ The PPI is easy to use and transparent in its accuracy for targeting at the individual level, which varies depending on the poverty cutoff selected. However, it encompasses several indicators that are unlikely to change over time, such as household size, and may not be very sensitive to monitoring economic change over the time frame of an average project.^4^ Furthermore, OVC programs are multidimensional and require tools that measure an expanded definition of vulnerability that comprises several dimensions of household and child health and well-being in addition to poverty.

Several OVC programs have approached this challenge by developing short scorecards with indicators related to program objectives. Because data are usually collected by community volunteers acting as case managers, the tools must be simple and easy to use for data collectors with limited education. This need, in addition to the large scale of many programs, places a premium on streamlining data collection efforts, so implementers often prefer a single tool to serve the functions of targeting, M&E, matching households to appropriate interventions, and determining readiness for graduation.

Economic strengthening implementers would prefer a single tool to serve the functions of targeting, M&E, matching households to interventions, and determining readiness for graduation.

**When these types of tools are scored and aggregated as an index measure of vulnerability, they can give implementers a false sense of accuracy and result in failure to target the neediest households.** An influential example of this kind of tool is the Vulnerability Assessment Tool (VAT) used by the Sustainable COmprehensive REsponses (SCORE) for Vulnerable Children and their Families project, implemented by AVSI, in Uganda.^5^ This tool includes questions on household assets and income, child protection, food security, parental status, basic services, and the enumerator's impression to classify households according to PEPFAR's categories of household economic status: destitute, struggling to make ends meet, or prepared to grow.^1^ The VAT was also the basis of the Vulnerability Index (VI) officially adopted by the Government of Uganda for OVC programs. However, a test of the VI demonstrated that it was not sufficiently sensitive to identify households with the most critical needs, limiting its utility as a targeting tool.^6^

The Government of Uganda has since adapted an updated version of the VAT called the Household Vulnerability Assessment Tool (HVAT). The HVAT collects information on child and household well-being including questions on economic status, health, water and sanitation, education, psychosocial support and basic care (of children), child protection, and legal support. Like the VAT, it generates a classification of households according to the 3 levels of vulnerability described in PEPFAR guidance. However, these categories are based on an arbitrary set of cut-off points based on score quartiles, and there is no clear theoretical basis for the system of weighting scores for either tool.

ASPIRES attempted to develop more accurate measures of economic vulnerability that could be used for OVC program targeting on 3 different occasions. In 2014, ASPIRES conducted a vulnerability assessment to inform economic strengthening intervention targeting for OVC programs in Côte d'Ivoire. In a review of the literature on vulnerability assessment methods for economic strengthening projects, ASPIRES found that few tools used by OVC programs had undergone any formal validation process. As such, ASPIRES implemented a household survey with the objective of developing a validated scale that could be used to target households at the individual level. In 2 other instances, ASPIRES did not attempt to develop vulnerability scales, but used a programmatic approach to explore the validity of M&E tools developed for different economic strengthening programs in Uganda and South Africa. This article describes the results from those efforts.

## THREE COUNTRY CASES: METHODS AND FINDINGS

### Côte d'Ivoire Assessment

We used a rigorous psychometric approach to develop an economic vulnerability scale in Côte d'Ivoire based on the Sustainable Livelihoods approach.^7^ We conducted a cross-sectional survey of 3,749 households in 5 health regions of Côte d'Ivoire in an attempt to develop a measure of USAID/Côte d'Ivoire's broad definition of vulnerability as the degree of inability of households to provide for the health, education, and nutritional needs of household members with and without HIV infection to mitigate the economic and health impact of HIV, cope with infection, and reduce their risk for acquiring HIV (for those without HIV).

The instrument was derived from existing survey tools to assess a comprehensive set of asset “capitals,” including financial, social, natural, financial, and physical capital, country-specific indicators from national household surveys, and formative qualitative data. In the absence of an existing validated measure of the broad definition of vulnerability that we attempted to measure, we used validated food security and poverty measures, which we expected to correlate with economic vulnerability, to test the validity of our tool. We used the PPI to measure poverty and the Food Consumption Score (FCS) and Reduced Coping Strategies Index (rCSI) to measure food security. Additionally, data collectors trained on the PEPFAR classifications of household status provided their own subjective assessments of each household as a fourth validation measure. Indicators were reviewed by a stakeholder advisory group and the survey was pretested and refined before data were collected.

Using principal component analysis (PCA), we attempted to identify sets of correlated vulnerabilities and derive a small number of composite scores (components) to create an index for targeting vulnerable households for enrollment into an economic strengthening program and matching them to appropriate interventions. We compared the mean component scores from the PCA to data collector classifications of household economic vulnerability using ANOVA. We also classified the households into 4 different vulnerability categories using distributions derived from a participatory vulnerability ranking activity.

We selected 65 of 98 variables in the PCA based on completeness and variability. Based on scree plot data, we retained 4 components but these 4 components explained only 21% of the total variance among the items. **This means that our measure explained only 21% of what distinguished households as more or less vulnerable based on the variables we included in our measure.** Although there is no minimum accepted threshold, a rule of thumb proposed for multivariate analysis in the social sciences is that a solution should explain roughly 60% of variance to be considered satisfactory.^8^ The first (and largest) component, which captured food security and wealth measures, explained only 8% of the variance. In other words, households classified at the same level of vulnerability could have vastly different sets of responses to the questions in our survey, suggesting that very different sets of variables could lead to the same economic status outcomes.

Overall, we were unable to reduce the variables for our broad construct of HIV-related economic vulnerability down to a scale. We concluded that there were many pathways to household economic vulnerability in our sample and that appropriate programmatic responses should be tailored to individual household needs using a case management approach rather than an economic classification based on a scale.

There are many pathways to household economic vulnerability, so programmatic responses should be tailored to individual household needs using a case management approach rather than an economic classification based on a scale.

### Uganda Assessment

ASPIRES' Family Care activity is managing 2 learning projects in Uganda to build evidence on how economic strengthening and social support can prevent unnecessary family–child separation and reintegrate separated families: Family Resilience (FARE), implemented by AVSI, and Economic Strengthening to Keep and Reintegrate Children into Families (ESFAM), implemented by ChildFund. In 2016, we worked with implementers to develop a short tool to assess the economic status of program beneficiaries for matching households to interventions and M&E purposes.

The HVAT is the basis for the tools used by both projects. Both programs modified the government tool to add indicators relevant to family–child separation and economic vulnerability: AVSI developing the FARE Household Vulnerability Assessment Tool (FARE HVAT) and ChildFund adapting the Family Status Vulnerability Index (FSVI) that it had developed for a related project. The scoring of these tools reflects these changes.

At the same time, Family Care developed a 9-item “Simple Economic Strengthening Tool” to generate common household economic classifications that could be used for cross-project comparison (Table 1). Family Care selected among existing economic indicators that were consistent across the FSVI and HVAT tools and that corresponded with the PEPFAR categories and LIFT (Livelihoods and Food Security Technical Assistance) livelihoods framework.^9^ An analysis of these frameworks showed 5 main domains of economic status:

1. Ability to pay for basic needs
2. Consistency/volatility of income
3. Availability of liquid assets and savings
4. Food security
5. Availability of assets to respond to shocks

**TABLE 1.** Simple Economic Strengthening Tool Developed for Cross-Project Comparisons in Uganda

**1.**	**What is the MAIN source of household income?**								
Options	a) None	b) Remittances, pension, gratuity, donations	c) Casual laborer	d) Informal job/employment	e) Peasantry farming/hiring out labour on other farms/garden	f) Petty business	g) Formal business	h) Commercial farming	i) Formal job/ employment
Score	3	2	2	2	2	0	0	0	0
**2.**	**What is the current monthly HH income? (express amount in Uganda Shillings, then score according to range)**								
__________________________ Uganda Shillings									
Options	a) Less than 50,000		b) 50,000 – 100,000		c) 100,000 –150,000	d) 150,000 – 200,000		e) Above 200,000	
Score	3		2		2	2		0	
**3.**	**How much money does the household have in savings?**								
__________________________ Uganda Shillings									
Options	a) Less than 30,000		b) 30,000 – 60,000		c) 60,000 –90,000	d) 90,000 –120,000		e) Above 120,000	
Score	3		2		2	1		0	
**4.**	**In how many of the last three months have you consistently been able to pay for the following items without having to sell HH productive assets like land, bicycle or borrowing at very high rates of interest (more than 30%)?**								
						**Number of months (0–3)**			
	1) Food, shelter, and water								
	2) Health care								
	3) Education								
	**Add total months (1 + 2+3)→**								
Options	a) Total=9		b) Total=8		c) Total=7	d) Total=4–6		e) Total=0–3	
Score	0		0		1	2		3	
**5.**	**If you had an unexpected shock, like a death in the family, happen tomorrow, how would you handle the expenses? (tick all that apply)**								
Options	(Do not read the options below; wait for the response and then tick those that correspond.)					Tick all that apply		Circle highest score	
	1) Pay with cash on hand/savings							0	
	2) Seek contributions/gifts from friends, relatives, community members, church help, etc.							3	
	3) Request help from a charitable organization, CBO, NGO							3	
	4) Borrow from a friend or relative or savings group and pay back later							1	
	5) Look for another source of income near my home							1	
	6) Reduce household spending a little							1	
	7) Reduce household spending a lot							2	
	8) Sell small livestock, household goods or items used in the household							2	
	9) Migrate for work							2	
	10) Borrow from money lender at high interest							3	
	11) Sell bicycle, land, tools or other items that help produce income							3	
	12) Break up the household—send children to others to care for							3	
	13) Go without food							3	
	14) Engage in transactional sex or illegal activities							3	
**6.**	**Over the past [12 months (baseline)/6 months (subsequent)], what has been the MAIN source of food consumed by your HH?**								
Options	a) Donated		b) Given in return for work only		c) Bought from the market			d) Home grown	
Score	3		3		2			0	
**7.**	**How many meals does the HH have in a day?**								
Options	a) Some days, no meal		b) One meal		c) 2 meals per day			d) 3 or more meals per day	
Score	3		3		1			0	
**8.**	**Do the following apply to this HH? Indicate (Yes/No) (observe for yourself where applicable)**						**Yes**	**No**	**N/A**
	1) Does the HH have access to safe water within 30 minutes (half an hour) or harvest rain water for domestic use?								
	2) Does the HH have a clean compound?								
	3) Does the HH have access to a public health facility within 5 kilometers?								
	4) Does the HH have a drying rack for HH utensils?								
	5) Does the HH have a garbage pit or dust bin?								
	6) Does the HH have a separate house for animals?								
	7) Does the HH have clean water and soap for hand washing?								
	8) Do all HH members sleep under a mosquito net?								
Options	a) If 4 or more are No		b) If 3 are No		c) If 2 are No	d) If 1 is No	e) If all are Yes or N/A		
Score	3		3		2	1	0		
**9.**	**Does the household have a stable shelter that is adequate, safe, and dry? (observe yourself)**								
Options	a) No stable shelter, adequate or safe place to live		b) Shelter is not adequate, needs major repairs		c) Shelter needs somerepairs but is fairly adequate, safe, and dry		d) Shelter is safe,adequate, and dry		
Score	3		2		2		0		

Abbreviations: CBO, community-based organization; HH, household.

To ensure that all domains were covered, Family Care had requested the 2 projects to add or harmonize some indicators across the FSVI and HVAT. A total of 9 questions were selected for the Simple Economic Strengthening Tool. Each was scored on a scale of 0–4, in accordance with the scoring systems already in place for the HVAT and FSVI. Individual question scores were used to generate a classification based on the PEPFAR descriptions of economic status (destitute, struggling, prepared to grow, or not vulnerable) for each domain. The 5 domain classifications were then used to determine the household classification based on an algorithm developed by Family Care. This complex scoring method was meant to account for the vulnerability dynamics implied by different combinations of scores across questions and domains.

Family Care then analyzed baseline data for 114 FARE target households using the Simple Economic Strengthening Tool. Due to poor correlation with the PPI, Family Care's selected validation measure, the scoring method was then revised based on implementer input so that each question was scored on a scale of 0–3, with scores corresponding to the PEPFAR categories as below:

- 0 points=not vulnerable
- 1 point=prepared to grow
- 2 points=struggling to make ends meet
- 3 points=destitute

Each question was weighted evenly, and final classifications were based on an unweighted average across all scores. The question on shocks originally had multiple responses with the same point value but was revised so that questions that previously had the same value now had different values. As such, Family Care was unable to convert the raw scores for this domain to the new scoring scheme and this domain was dropped from the revised tool. Four of the original 5 domains were retained, with 2 questions each, so domain scores were evenly weighted. FARE used the Simple Economic Strengthening Tool scoring method during baseline data collection among 114 households.

Because poverty likelihood, as measured using the PPI, was expected to vary in the same direction as economic vulnerability as measured by the Simple Economic Strengthening Tool, the PPI was conducted simultaneously with all households as a validation measure. Among the poverty line calculations provided by the PPI, Family Care selected the US$2/day poverty line calculated at 2005 Purchasing Power Parity (PPP), which is the most updated measure of PPP. The correlation between the revised Simple Economic Strengthening Tool scores and PPI-calculated poverty likelihoods at the $2/day level increased to a low, positive correlation (r=.43). Though there is no single accepted method for interpreting correlation coefficients, a common rule of thumb would suggest that a moderate correlation should reach at least .50.^10^

Although the Simple Economic Strengthening Tool contained useful indicators for program monitoring, its ultimate output was only moderately correlated with our poverty measure, which also casts doubt on the validity of the economic vulnerability classifications it generated.

### South Africa Assessment

Structural economic drivers at the household and individual levels play a major role in driving disproportionate rates of HIV infection among adolescent girls in South Africa.^11^ PEPFAR program implementers using economic strengthening interventions to address these drivers need to monitor progress with high-quality M&E tools. Although there are some existing measures of adolescent girls' vulnerability to HIV, none focus specifically on the pathway between individual economic status and HIV risk. The purpose of this assessment was to develop and validate M&E survey tools to assess economic vulnerability for households and individual girls participating in socioeconomic interventions offered by the South African NGO Children in Distress Network (CINDI) to enhance resilience against HIV.

Between October 20 and December 7, 2016, 87 individual interviews with girls and 93 household-level interviews were conducted among a sample of girls aged 10–19 participating in CINDI programs and their caregivers.

The household-level tool (Household Tool) was developed using the same domains derived from the PEPFAR guidance as the Simple Economic Strengthening Tool, with similar questions. Each question was scored 0–3 points and averaged to generate domain scores. Domain scores were then averaged to generate a total score, which was rounded to the nearest whole number to generate a classification. Validity measures included the PPI, household rankings derived from participatory exercises, and subjective classifications generated by the data collector and the respondent.

Thirteen items for the individual-level tool (Girl Tool) were derived from a review of the literature on the links between economic status and HIV for adolescent girls in sub-Saharan Africa based on an assessment of potential variability and on consultation with implementers and an outside expert in health and economic empowerment programs for adolescent girls. Each item was equally weighted, with each indicator scored at a range of 0–3, for an overall range of 0–39. The Vulnerable Girl Index, a measure of adolescent girls' HIV vulnerability validated in several Southern African countries,^12^ was used as a validity measure for the Girl Tool. Structured interviews were conducted with 4 program staff members to assess the acceptability, feasibility, and perceived validity of the Household and Girl Tools.

Neither the Household Tool nor the Girl Tool could be validated using the selected validation measures, and both tools had low inter-item reliability (α=.45 and .21, respectively). We also found that the VGI scores accounted for little variance in the data collected and had poor inter-item reliability (α=.19), where a typical benchmark for high inter-item reliability is at least .70.^13^

Table 2 summarizes the 4 ASPIRES vulnerability tools and assessments, including the definition of economic vulnerability, domains assessed, validation measures, and findings.

**TABLE 2.** Summary of ASPIRES Assessments of Economic Vulnerability Tools

Tool	Definition of Economic Vulnerability	Domains Assessed	Validation Measures	Findings
Côte d'Ivoire Vulnerability Assessment	The degree of inability of households to provide for the health, education, and nutritional needs of household members to mitigate the economic and health impact of HIV, cope with infection, and reduce their risk for acquiring HIV (for those without HIV).	Financial capital Physical capital Natural capital Social capital Human capital	Poverty likelihood Côte d'Ivoire Progress out of Poverty Index (PPI)	The 4 components created using principal component analysis explained only 21% of the variance among items Component 1 was moderately correlated (r=.69) with the rCSI, FCS (r=.55), and PPI (r=.46) The 65 vulnerability measures examined did not cluster in ways that would allow for the creation of a small number of composite measures to develop a scale
			Food Security: Reduced Coping Strategies Index (rCSI) Food Consumption Score (FCS)	
Uganda Simple Economic Strengthening Tool	PEPFAR classifications of: Destitute Struggling to make ends meet Prepared to grow Not vulnerable	Ability to pay for basic needs Consistency/volatility of income Availability of liquid assets and savings Food security Availability of assets to respond to shocks	Poverty likelihood Uganda Progress out of Poverty Index (PPI)	Moderate, positive correlation with poverty likelihood (r=.43)
South Africa Household Tool	PEPFAR classifications of: Destitute Struggling to make ends meet Prepared to grow Not vulnerable	Ability to pay for basic needs Consistency/volatility of income Availability of liquid assets and savings Food security Availability of assets to respond to shocks	Poverty likelihood South Africa Progress out of Poverty Index (PPI)	No significant association between poverty likelihood and tool classification (*P*=.25) No significant association between classifications generated during community ranking exercise and tool classification (*P*=.77) Modest association between self-classification and tool classification (weighted kappa=.32) Significant but non-linear association between data collector classification and tool classification (*P*=.003)
			Local classifications Community rankings Self-classification Data collector classification	
South Africa Girl Tool	The prevalence of economic factors that lead to transactional sex, and therefore increase risk for HIV.	Perception of needs met Pressure to contribute to the household Availability of cash Food security Shocks Safety nets Financial goals Control over assets Control over economic decision making Personal documentation Gender attitudes	Adolescent girls' HIV vulnerability Vulnerable Girls Index (VGI)	No statistically significant correlations between the Girl Tool and the VGI (*P*=.25)

Abbreviations: ASPIRES, Accelerating Strategies for Practical Innovation and Research in Economic Strengthening; PEPFAR, U.S. President's Plan for Emergency AIDS Relief.

## DISCUSSION

In Côte d'Ivoire, Uganda, and South Africa, ASPIRES hoped to develop simple, valid tools to measure economic vulnerability for economic strengthening program targeting, intervention matching, and M&E. Three of the 4 tools developed focused on classifying households according to PEPFAR categories, while 1 tool focused on measuring individual-level economic vulnerability among adolescent girls. The 3 country cases demonstrate the challenge in reducing broad constructs of economic vulnerability into simple indices to classify households in a way that accounts for a substantial amount of variance between households at locally defined vulnerability levels. None of the measures varied significantly with their validation items, which included poverty measures derived from country-specific PPI scorecards.

The 3 country cases demonstrate the challenge in reducing broad economic vulnerability constructs into simple indices.

### Quantifying and Classifying Economic Status

These findings do not mean that the tools cannot be useful for M&E of economic strengthening programs; each tool captures at least some element of a broader construct of economic vulnerability that is useful for measuring change. However, the Côte d'Ivoire and South Africa assessment measures did not explain most of the variation between households, and none of the measures explained what distinguishes households from one another according to validation measures, including community definitions of what makes a household less likely to withstand economic shocks. As such, these measures may be helpful for measuring some of the factors that make households and individuals economically vulnerable, but they are likely not able to capture most of the many pathways that lead to negative economic outcomes. **This means that the 3 measures are not predictive of negative economic outcomes, making them poor options for *targeting* tools.**

The tools may be helpful for measuring some of the factors that make households and individuals economically vulnerable, but they are likely not able to capture most of the pathways that lead to negative economic outcomes.

Other tools that have sought to quantify broad constructs of vulnerability have faced similar difficulties. For example, a validation study of MEASURE Evaluation's Child Status Index (CSI) concluded that the tool was not a valid measure of child-level vulnerability in rural Malawi,^14^ prompting MEASURE Evaluation to release documentation clarifying the role of the tool. MEASURE Evaluation recommends using the CSI for case management and monitoring, but cautions against aggregating scores across the indicators to generate a single score or using it for evaluation or targeting.^15^

**For narrower definitions of vulnerability, the accuracy of existing tools may be highly context-dependent.** As a validation measure for the Girl Tool in South Africa, the VGI was the only tool found in our literature review that measured HIV-related vulnerability at the individual level for adolescent girls. Though validated for several southern African countries, it had not been validated for South Africa. In our assessment, the VGI had very low inter-item reliability, meaning it did not explain much of the variance in our sample. Our experience with the VGI highlights the challenge of using validated instruments in contexts where they have not been validated.

### Targeting for Program Inclusion

MEASURE Evaluation has developed a separate targeting tool for OVC programs in Uganda called the Household Vulnerability Prioritization Tool (HVPT), which has been adapted for several other country contexts. This approach essentially identifies “red flags” for negative outcomes, and prioritizes households with these characteristics. FARE used a version of this tool to identify and enroll beneficiaries. It does not assign a point value to these indicators like a scale. Rather, potential participants are selected into a program based on the presence of indicators that are divided into 3 tiers based on severity and what the program most intends to impact. The HVPT developed for Uganda uses a 3-step prioritization process. Households with a child protection issue are prioritized first. Next, the HVPT prioritizes households with “high vulnerability” indicators, including: (1) child-headed households, (2) households with any child not eating for a 24-hour period in the last month, (3) households with individuals living with HIV, and (4) households where at least 1 child is not in school. The third set of households prioritized is based on the number of vulnerability domains where the household is considered vulnerable. This tool was developed in consultation with stakeholders to define vulnerability and identify the most important characteristics associated with households in the greatest need of services.^16^

A targeting tool developed by MEASURE Evaluation allows selection of program participants based on the presence of “red flag” indicators associated with negative economic outcomes, rather than assigning a point value to the indicators like a scale.

“Red flag” indicators can be identified by analyzing large datasets to identify which characteristics are most associated with negative outcomes of programmatic interest. For example, UNICEF analyzed household survey data across 11 countries with a high HIV prevalence to identify factors most associated with negative outcomes for children.^17^ It found that poverty; not living with either parent; losing one or both parents; or living in a household with adults with no education were most associated with negative outcomes. These characteristics can serve as targeting criteria for vulnerable children.

Another way to identify red flags for targeting is to analyze poverty dynamics using national household survey data. There are several common econometric methods that can be used for this.^2^ Recently, ACDI/VOCA's Leveraging Economic Opportunities (LEO) project used this approach to conduct a series of studies on “sustained poverty escapes” using panel data and life history interviews to identify characteristics associated with falling below the poverty line, hovering near the poverty line, or sustainably escaping poverty over several years.^18^ For example, in Bangladesh, households that had a higher dependency ratio, less livestock, and less cultivable land were more likely to experience only temporary escapes from poverty over time.^19^ These indicators shed light on characteristics that lead to vulnerability or resilience, and can be used to target vulnerable households and set program benchmarks. **Rather than aggregating scores across indicators associated with vulnerability, “red flag” approaches can be used to prioritize households with characteristics most associated with negative outcomes to make sure that households with the greatest need are included in a program.** This bypasses the problem of trying to capture many pathways of vulnerability in a single tool. As with any measurement tool, implementers should attempt to validate “red flag” indicators before using them to prioritize households for program enrollment.

### Limitations

The 3 country cases included here have several limitations. Since there is no direct, “gold standard” measure of vulnerability available to validate any of the tools we tested, we used other validated measures we expected to vary in the same direction as economic vulnerability, including measures of poverty, food security, and local perceptions of vulnerability. Our poverty measure, the PPI, is an efficient and validated tool but subject to error at the individual level. Our food security measures, the rCSI and FCS, have both been shown to correlate with poverty measures but have sensitivity limitations.^20^ Though local perceptions are commonly used in poverty targeting,^21^ we trained enumerators in Côte d'Ivoire and South Africa to classify households based on the PEPFAR categories of economic status, which have not been validated, and their perceptions were subjective.

In light of these limitations, although our Côte d'Ivoire study employed rigorous methods to develop a scale, the scale could not be fully validated. Our survey tool was based on a complex definition of economic vulnerability for which we did not have any direct measures that could be used to validate our measurement model. As a result, we relied on the face validity of the measures included in the PCA analyses to validate the measure, in addition to examining correlations of the components with the well-established indices mentioned above.

On the other hand, neither the Simple Economic Strengthening Tool in Uganda nor the South Africa assessment were developed using rigorous scale development methods, but rather were developed as programmatic tools to track participant progress. As such, the household-level tools used in Uganda and South Africa equally weight the domains analyzed, which assumes each domain has an equal effect on vulnerability. Additionally, although the Côte d'Ivoire study enjoyed a robust sample size, the Uganda and South Africa assessments had very low sample sizes, which limits our ability to statistically analyze and draw conclusions from them.

The South Africa assessment was also affected by problems with the participatory ranking exercises. To get a good representation of participant households in our ranking data, we planned to survey girls where program participants were concentrated in a single neighborhood of 50 households that knew each other, then conduct participatory wealth ranking exercises in the same communities. Instead, households on the participant rosters were spread across communities, making it difficult to conduct focus groups with a large number of participant households represented. Only 14 participant households received rankings in the focus groups, limiting our ability to draw any conclusions by comparing the Household Tool rankings to the rankings generated in the focus groups for these households.

## CONCLUSION

Economic strengthening programming addresses the economic drivers of HIV and negative child well-being outcomes, so economic status is an important intermediate outcome that implementers must measure. At the same time, programs implementing economic strengthening interventions are under pressure to identify and intervene with the most vulnerable households. Many have attempted to combine the functions of targeting, monitoring, and evaluation into a single tool by quantifying broad constructs of vulnerability into a simple index. ASPIRES' recent experiences in Côte d'Ivoire, South Africa, and Uganda suggest that such simple indices may not accurately capture a broad construct of vulnerability and are not accurate for targeting. While the sample sizes for the South Africa and Uganda assessments are insufficient to draw firm conclusions, the trends in our analysis across the 3 country cases demonstrate that although several tools are used by implementers to measure household-level economic vulnerability, there is little evidence that these tools are measuring what they are intended to measure—that is, a household's susceptibility to negative economic outcomes. We recommend that researchers and implementers focus on developing M&E instruments to capture narrower definitions of vulnerability based on characteristics their programs intend to affect. We also recommend using separate tools for targeting based on context-specific “red flag” indicators with evidence-based links to negative outcomes, rather than potentially specious scales that attempt to summarize broad constructs of vulnerability. Finally, policy makers and donors should avoid reliance on simplified metrics of economic vulnerability in the development programs they support, as these may falsely categorize participants and leave the most vulnerable out of an intervention.

Policy makers and donors should avoid relying on simplified metrics to economic vulnerability, as they may leave the most vulnerable out of an intervention.

## Notes

**Peer Reviewed**

**First Published Online:** March 1, 2018

**Funding:** USAID.

## References

[1] U.S. President's Emergency Plan for AIDS Relief (PEPFAR). Guidance for Orphans and Vulnerable Children Programming. Washington, DC: PEPFAR; 2012. https://www.pepfar.gov/documents/organization/195702.pdf. Accessed January 17, 2018.

[2] Hoddinott J Quisumbing A. Methods for microeconometric risk and vulnerability assessments. Social Protection Discussion Paper No. 0324. Washington, DC: World Bank; 2003. http://documents.worldbank.org/curated/en/948651468780562854/Methods-for-microeconometric-risk-and-vulnerability-assessments. Accessed January 17, 2018.

[3] Schreiner M. A simple poverty scorecard for Côte d'Ivoire. 2013. http://www.microfinance.com/English/Papers/Scoring_Poverty_Cote_dIvoire_2008_EN.pdf. Accessed January 17, 2018.

[4] Desiere S Vellema W D'Haese M. A validity assessment of the Progress out of Poverty Index (PPI). Eval Program Plann. 2015;49:10–18. 10.1016/j.evalprogplan.2014.11.002. 25462937

[5] Lowicki-Zucca M Walugembe P Ogaba I Langol S. Savings groups as a socioeconomic strategy to improve protection of moderately and critically vulnerable children in Uganda. Child Youth Serv Rev. 2014;47:176–181. 10.1016/j.childyouth.2014.08.011

[6] Cannon M. Uganda Vulnerability Index assessment results. Chapel Hill, NC: MEASURE Evaluation; 2014. https://www.measureevaluation.org/resources/publications/sr-14-93. Accessed January 17, 2018.

[7] Burke HM Moret W Field S Chen M Zeng Y Seka FM. Assessing household economic vulnerability in HIV-affected communities in five regions of Côte d'Ivoire. PLoS One. 2016;11(9):e0163285. 10.1371/journal.pone.0163285. 27655530 PMC5031437

[8] Hair J Black W Babin B Anderson R. Multivariate Data Analysis: A Global Perspective. 7th ed. New Jersey: Pearson Ed.; 2010.

[9] Woller G. Livelihood and food security conceptual framework. Washington, DC: Livelihood and Food Security Technical Assistance (LIFT); 2011. http://theliftproject.org/wp-content/uploads/2013/03/Livelihood-and-Food-Security-Conceptual-Framework.pdf. Accessed January 17, 2018.

[10] Hinkle DE Wiersma W Jurs SG. Applied Statistics for the Behavioral Sciences. Houghton Mifflin; 2003.

[11] Dellar R Waxman A Abdool Karim Q. Understanding and responding to HIV risk in young South African women: clinical perspectives. S Afr Med J. 2015;105(11):952-952. 10.7196/SAMJ.2015.v105i11.10099. 26937509

[12] Underwood CR Schwandt HM. Assessing girls' HIV vulnerability: evidence from Botswana, Malawi and Mozambique. Health Policy Plan. 2016;31(6):729–735. 10.1093/heapol/czv123. 26701916

[13] Tavakol M Dennick R. Making sense of Cronbach's alpha. Int J Med Educ. 2011;2:53–55. 10.5116/ijme.4dfb.8dfd. 28029643 PMC4205511

[14] Sabin L Tsoka M Brooks MI Miller C. Measuring vulnerability among orphans and vulnerable children in rural Malawi: validation study of the Child Status Index tool. J Acquir Immune Defic Syndr. 2011;58(1):e1–e10. 10.1097/QAI.0b013e3182254298. 21654501

[15] MEASURE Evaluation. Clarification regarding usage of the Child Status Index. Chapel Hill, NC: MEASURE Evaluation; 2012. https://www.measureevaluation.org/our-work/ovc/clarification-regarding-usage-of-the-child-status-index. Accessed January 17, 2018.

[16] A purpose-built tool provides transparency for enrollment in services. MEASURE Evaluation website. https://www.measureevaluation.org/news/a-purpose-built-tool-provides-transparency-for-enrollment-in-services. Accessed January 17, 2018.

[17] UNICEF. Measuring the Determinants of Childhood Vulnerability. New York: UNICEF; 2014. https://data.unicef.org/wp-content/uploads/2015/12/Measuring-the-Determinants-of-Childhood-Vulnerability_Final-Report-5_8-LR-_172.pdf. Accessed January 17, 2018.

[18] Overseas Development Institute (ODI). Resilience and sustained escapes from poverty: highlights from research in Bangladesh, Ethiopia and Uganda. 2016. https://microlinks.org/library/resilience-and-sustained-escapes-poverty-highlights-research-bangladesh-ethiopia-and-uganda. Accessed January 17, 2018.

[19] Scott L Diwakar V. Ensuring escapes from poverty are sustained in rural Bangladesh. Leveraging Economic Opportunities Report No. 32. 2016. https://www.odi.org/publications/10536-ensuring-escapes-poverty-are-sustained-rural-bangladesh. Accessed January 17, 2018.

[20] Jones AD Ngure FM Pelto G Young SL. What are we assessing when we measure food security? A compendium and review of current metrics. Adv Nutr. 2013;4(5):481–505. 10.3945/an.113.004119. 24038241 PMC3771133

[21] Devereux S Masset E Sabates-Wheeler R Samson M te Lintelo D Rivas A-M. Evaluating the targeting effectiveness of social transfers: a literature review. IDS Working Paper 460. Brighton, UK: Institute for Development Studies; 2015. https://www.ids.ac.uk/publication/evaluating-the-targeting-effectiveness-of-social-transfers-a-literature-review. Accessed January 17, 2018.

